# Prevalence and predictors of food insecurity in conflict zones among displaced families in Lebanon during the war: a cross-sectional study

**DOI:** 10.3389/fpubh.2025.1648552

**Published:** 2025-11-20

**Authors:** Paula Hage Boutros, Samah Hachem, Rola Bou Serhal, Noura Yazbeck, Firas Azzam, Bachir Atallah

**Affiliations:** 1 Faculty of Health Sciences, Department of Nutrition, Modern University for Business and Science, Beirut, Lebanon; 2 Institut National de Sante Publique, d’Epidemiologie Clinique et de Toxicologie-Liban (INSPECT-LB), Beirut, Lebanon; 3 Department of Public Health, Modern University for Business and Science, Beirut, Lebanon; 4 Department of General Education, Chamberlain University, Troy Campus, MI, United States; 5 Department of Research, Modern University for Business and Science, Beirut, Lebanon; 6 Faculty of Health Sciences, Department of Nursing, Modern University for Business and Science, Beirut, Lebanon

**Keywords:** food insecurity, mental health, displacement, PTSD, Lebanon

## Abstract

**Background:**

The September–December 2024 conflict in Lebanon resulted in the displacement of over 1.5 million individuals, compounding the country’s existing economic and humanitarian crises. This study is aimed at assessing the prevalence and predictors of food insecurity (FI) among displaced families, focusing on key socioeconomic and psychological determinants.

**Methods:**

A cross-sectional survey was conducted among 400 displaced households across Lebanon. Data were collected on demographics, food security (Arab Family Food Security Scale), malnutrition (MUAC), income, education, household size, and mental health indicators including depression (PHQ-2), anxiety (GAD-2), PTSD (PCL-5), and resilience (BRS). Logistic regression identified predictors of FI.

**Results:**

FI affected 42.4% of households, with 16.1% experiencing very low food security and only 28% fully food secure. Male participants reported higher food security than females (67% vs. 54.5%, *p* = 0.028). Larger families and monthly income < $700 (83.3% of sample) were significantly associated with FI (*p* = 0.003 and *p* < 0.001, respectively). Malnutrition prevalence was 7.2%, while obesity was 21%, reflecting a dual burden. Mental health distress was significantly higher among the food insecure: depression (3.91 vs. 2.56), anxiety (4.03 vs. 2.51), and PTSD (48.7 vs. 34.6); all *p* < 0.001. Resilience scores showed no association with FI (*p* = 0.106). Logistic regression identified low income (OR = 0.224), depression (OR = 2.099), anxiety (OR = 1.864), PTSD (OR = 1.023), and food aid (OR = 1.732) as significant FI predictors.

**Conclusion:**

Displaced Lebanese families face high rates of food insecurity linked to economic vulnerability and mental distress. While aid reduces malnutrition, it falls short in addressing FI. Integrated policies targeting income, mental health, and systemic aid delivery are essential for improving resilience and nutritional outcomes in crisis settings.

## Introduction

Food insecurity (FI), defined as the lack of consistent access to sufficient, safe, and nutritious food, is a pressing global issue, particularly in conflict zones (1) Food security, on the other hand, is defined as the state where people can physically, socially, and economically access sufficient food that will enable them to lead an effective and healthy life. Conflict-affected regions, the risks of FI are amplified as agricultural production, food distribution systems, and livelihoods are disrupted. Wars and armed conflicts not only destroy infrastructure but also lead to mass displacement, inflation, and rising unemployment, all of which undermine food availability and access (2). The World Food Programme (WFP) has reported that in conflict zones, households are up to three times more likely to experience FI compared with stable settings, underscoring the scale of the challenge (3).

Lebanon has had a long-standing issue of FI due to recurrent crises. The Lebanese Civil War (1975–1990) disrupted agricultural production, increased import dependence, and raised food costs (4). Similarly, the 2006 war with Israel damaged supply chains and infrastructure, aggravating food scarcity and disproportionately affecting vulnerable households (5). More recently, the country’s economic collapse since 2019 has been described as one of the world’s worst financial crises since the mid-19th century (6). The September–December 2024 war further compounded the crisis, displacing over 1.5 million individuals, primarily from southern Lebanon and parts of the Bekaa Valley. Current estimates suggest that more than 1.65 million people—both citizens and refugees—are food insecure (7), with levels comparable to Syria and Yemen, where armed conflicts have devastated agriculture and food supply chains (8).

Displacement exacerbates FI by disrupting livelihoods, severing access to stable income, and forcing reliance on humanitarian aid. Globally, approximately 80% of displaced persons face acute FI, with internally displaced populations being especially vulnerable due to limited protections and inadequate aid coverage (9). In Lebanon, internally displaced Lebanese families during the 2024 conflict were heavily dependent on overstretched public services, host communities, and intermittent relief distribution (10). Socioeconomic vulnerabilities such as unemployment, low income, and lack of financial stability further restricted food access, while structural barriers such as inflation, food price volatility, and weakened governance—exacerbated household fragility. Inconsistencies in aid delivery further reduced the effectiveness of interventions (11).

FI in Lebanon intersects with broader nutritional and health challenges. The country faces a “double burden of malnutrition,” where undernutrition and micronutrient deficiencies coexist with rising rates of overweight and obesity (12). This paradox reflects both reduced access to diverse, nutrient-rich foods and the increased reliance on cheap, calorie-dense products during crises. For displaced families with limited resources, food quantity often takes precedence over quality, heightening the risks of both malnutrition and diet-related chronic diseases (13).

Beyond physical health, FI has profound psychological consequences. Studies consistently link FI to stress, depression, anxiety, and post-traumatic stress disorder (PTSD) (14). In conflict and displacement contexts, these effects are compounded by trauma, uncertainty, and chronic insecurity. For parents in particular, the inability to provide food can generate feelings of shame, helplessness, and heightened distress. Households may adopt harmful coping mechanisms—reducing meal frequency, selling assets, resorting to child labor, or early marriage—to manage food scarcity (15). Although resilience is often considered a protective factor, evidence on its moderating role remains inconsistent, particularly in displaced populations (16).

Despite the urgency of the issue, research examining FI among displaced Lebanese households remains scarce. Much of the existing evidence on FI and mental health comes from high-income, Global North settings, where strong social protection systems, relatively stable markets, and institutional supports differ fundamentally from fragile contexts such as Lebanon. Findings from those settings may not directly apply, given Lebanon’s weak governance, reliance on humanitarian aid, and repeated cycles of crisis. Moreover, international reports often aggregate Lebanese citizens with refugee populations, obscuring the unique vulnerabilities of internally displaced Lebanese. Very few studies have disaggregated these groups to examine their distinct experiences, leaving policymakers with limited context-specific data to guide interventions.

Addressing this gap is critical. By focusing on internally displaced Lebanese families, this study generates new evidence on the prevalence of FI and its socioeconomic, nutritional, and psychological predictors in a conflict-affected setting. Unlike prior research that aggregates displaced populations, this study centers specifically on Lebanese households uprooted by the 2024 war. In doing so, it provides context-specific insights that can inform humanitarian strategies and longer-term resilience-building.

Therefore, this study aims to assess the prevalence of FI and identify its socioeconomic, nutritional and psychological predictors among displaced Lebanese families during the September–December 2024 war. By focusing on a nationally representative sample of internally displaced Lebanese households, this study contributes context-specific evidence that can inform humanitarian strategies and long-term resilience-building in conflict-affected regions.

## Materials and methods

### Consent to participate declaration

All participants were informed about the study’s purpose and procedures prior to their involvement. It was clearly stated that by proceeding with the survey, they were providing their consent to participate. This study was conducted in accordance with the ethical principles of the Declaration of Helsinki and was approved by the Ethics Committee at the Modern University of Business and Science (reference number: MU-20241106-49).

Participation was voluntary, and participants were told to withdraw at any time without any consequences.

### Study design

This study is a cross-sectional population survey conducted among N = 400 participants in different regions in Lebanon, specifically in Chouf, Aley, Hasbaya, and Rashaya areas, where the displaced families from the South and Bekaa areas had relocated. Data was collected from November to December 2024 across 20 public schools, which provided shelter for the displaced families. A total of 20 participants were recruited from each school using a simple random sampling strategy, ensuring that all eligible individuals had an equal chance of being selected. The overall response rate was approximately 85%. All participants were Lebanese citizens who had been internally displaced within Lebanon due to the 2024 war. No Syrian refugees were included. As no formal registry or screening system exists in Lebanon for internally displaced persons, displacement status was determined based on participants’ self-reported relocation following the conflict. The choice of displaced Lebanese individuals as the subject population is grounded in several reasons, including their high vulnerability and risk of malnutrition and mental health challenges.

Study inclusion criteria involved adults aged 18 and above, living in Lebanon, and both genders were included. People who lived abroad or fled the war to other countries were excluded, alongside those who did not consent to participate in the study. The participants’ selection did not consider any specific nutritional status or dietary pattern. The sample size was calculated using EpiInfo 7 software for population surveys. Since the approximate FI rate was unknown, an expected frequency of 50% was used, alongside a 5% margin of error, and a minimum sample of 384 was calculated (Epiinfo™, version 7.2.5.0).

### Data collection

All participants provided written informed consent prior to completing the survey. The consent form was embedded at the beginning of the Google Forms survey, and participants indicated their agreement by clicking “I agree to the above.” Adult participants were then interviewed by trained researchers who completed the online survey on their behalf to minimize potential recording errors. The survey consisted of several sections. The first included sociodemographic variables: parents’ age, gender, education level, family size, current and previous location, displacement duration, and monthly household income level. The parents’ weight, and mid-upper arm circumference (MUAC) were also measured.

Both self-reported participant responses and research-entered data were recorded on the Google form survey. The consent form clearly stated that participation was voluntary and that declining to participate would have no negative consequences. The questionnaire was available in both English and Arabic, and participants could choose their preferred language. The survey tool was used with the authors’ consent.

### Clinical measurements

#### Anthropometrics

Height was self-reported by participants. Weight was measured using a digital scale by the trained researchers after being calibrated following each data collection. BMI was calculated according to the formula: BMI = weight (kg) / (height (m)) ^2. BMI categories were defined based on Institute of Medicine (IOM) guidelines: Underweight: BMI < 18.5 kg/m^2^, normal weight: 18.5–24.9 kg/m^2^, overweight: 25.0–29.9 kg/ m^2^, and obese: ≥30.0 kg/m^2^ (17).

Mid-Upper Arm Circumference (MUAC) was assessed using a non-stretchable measuring tape on the left arm, at the midpoint between the acromion and olecranon process, with the arm relaxed. A MUAC of **<**23 cm was used as the cut-off for under-nutrition for adults (18).

#### Biochemical assessment

A finger-prick test was conducted using a portable glucometer to measure capillary blood glucose levels. Standard aseptic techniques were followed for all procedures. Participants were asked to report the fasting hours before testing to differentiate between fasting and random glucose measurements. Blood glucose levels were categorized according to the American Diabetes Association (ADA) criteria. For fasting blood glucose (defined as ≥8 h of fasting), values <5.6 mmol/L were considered normal, 5.6–6.9 mmol/L were classified as prediabetes, and values ≥7.0 mmol/L were considered indicative of diabetes. For random blood glucose measurements (taken at any time of day, regardless of the last meal), a value ≥11.1 mmol/L accompanied by symptoms of hyperglycemia was considered diagnostic of diabetes (19). For this study, we classified blood glucose values as either normal (<5.6 mmol/L) or elevated (≥5.6 mmol/L). It is important to note that glucose testing was included as an exploratory measure and was not part of the primary study aims.

#### Clinical variables

##### Food security

Food Security Assessment was done using the Arab Family Food Security Scale (AFFSS), validated in Lebanon, and showed good reliability and psychometric properties (20). Scores were initially classified into three categories: high food security (0–2), low food security (3–5), and very low food security (5–7). For the purposes of regression analysis, the low and very low categories were combined into a single “low food security” group (3–7) and compared against the “high food security” group (0–2).

*Depression:* Depression was screened using the two-item Patient Health Questionnaire (PHQ-2), a validated tool with a sensitivity of 83% and a specificity of 92% at a cut-off value of 3. It consists of two Likert-scale questions with answers ranging from “not at all” to “nearly every day,” coded 0 to 3 (21). The Arabic translation of the Patient Health Questionnaire was also previously validated (22).

##### Anxiety

Anxiety was screened using the Generalized Anxiety Disorder Scale (GAD-2), a validated tool that showed a 76% sensitivity and 81% specificity at a cut-off value of 3. The tool consists of two questions that rate the frequency of feeling nervous, anxious or on the edge, and that of not being able to stop or control worrying, and the answers to each item are coded from 0 to 3, with a maximum total score of 6 per respondent (23). The Arabic version of the Generalized Anxiety Disorder is validated (24).

##### Post-traumatic stress disorder

Trauma was screened using the Posttraumatic Stress Disorder Checklist for DSM-5 (PCL-5), a validated tool with good psychometric properties that consists of 20 items with Likert-scale answers coded 0 to 3 each, and higher scores indicating higher trauma levels (25). A PCL-5 score of 31 or higher was used to define PTSD cases. The Arabic version of the PCL-5 was validated in the Lebanese population (26).

##### Resilience

Resilience, defined as the ability to bounce back from stressful situations, was evaluated using the Brief Resilience Scale, a six-item tool with three items worded positively and three worded negatively (27). Responses are recorded on a Likert scale varying from 1 to note “strongly agree” to 5 corresponding to “strongly disagree.” Low resilience was defined by a BRS score less than 3, normal resilience was considered when the BRS score was between 3 and 4.30, and high resilience was defined by a BRS score higher than 4.30. The tool is validated in Arabic (28).

The final section consisted of additional questions, including receiving any psychological counseling during displacement, or receiving any assistance or support regarding food, to control for confounders.

### Statistical analysis

Data was analyzed using statistical software SPSS version 26. Descriptive analysis was used to represent the variables, where frequencies and percentages represented the qualitative variables, while continuous variables were represented by means, standard deviation, minimum, and maximum. The normality testing was checked graphically via histogram and QQ-Plot. The association between food security (High, Low) was tested with all the study variables (demographics, socioeconomic characteristics, mental health variables, and resilience). Chi-square test and independent t-test were used in the bivariate analysis. To assess the factors predicting low food security, a binary logistic analysis model was employed, including all the variables that were associated in the bivariate settings with *p* < 0.2. Standard assumptions for logistic regression, including assessment of multicollinearity among independent variables, were checked and no violations were detected. The survey was designed to require completion of all sections, so there was no missing data and no imputation was necessary. Statistically significant associations were set at 5% (*p* < 0.05).

## Results

Table 1 describes the baseline characteristics of the study population. A total of 400 participants were recruited, predominantly female (74.3%), with a mean age of 42.7 ± 15.4 years (range 18–95). Most participants had low educational attainment (27.3% no formal education; 36.3% primary; 14.5% secondary; 22.0% tertiary). The mean household size was 5.0 ± 2.6 members, including on average one child under 18. Households reported an average displacement duration of 55 months. Participants were mainly from Mount Lebanon (43.3%), Bekaa (24.3%), and South Lebanon (12.3%), with smaller proportions from Nabatiyeh (15.5%), Beirut (3.5%), Baalbeck-Hermel (0.3%), North Lebanon (0.3%), and Akkar (0.3%). Most participants (83.3%) reported a monthly household income below $700 (Table 1).

**Table 1:** Baseline characteristics of the study population (*N* = 400).

Variable	Category/Value	Frequency (%)/Mean ± SD^a^
Demographics		
Gender	Female	297 (74.3%)
	Male	103 (25.8%)
Age, years	Mean ± SD	42.65 ± 15.39
Household size	Mean ± SD	5.02 ± 2.58
Children under 18	Mean ± SD	1.00 ± 1.61
Displacement duration	Mean ± SD (months)	55.71 ± 33.82
Education level	No formal education	109 (27.3%)
	Primary education	145 (36.3%)
	Secondary education	58 (14.5%)
	Tertiary education	88 (22.0%)
Governorate (Current)	Mount Lebanon	173 (43.3%)
	Bekaa	97 (24.3%)
	South Lebanon	49 (12.3%)
	Nabatiyeh	62 (15.5%)
	Beirut	14 (3.5%)
	Baalbeck-Hermel	1 (0.3%)
	North Lebanon	1 (0.3%)
	Akkar	1 (0.3%)
Residence (Pre-displacement)	Nabatiyeh	153 (38.3%)
	South Lebanon	153 (38.3%)
	Mount Lebanon	41 (10.3%)
	Bekaa	21 (5.3%)
	North Lebanon	15 (3.8%)
	Akkar	1 (0.3%)
	Baalbeck-Hermel	1 (0.3%)
	Beirut	51 (12.8%)
Monthly household income	< $700	333 (83.3%)
	$700 - $1,500	57 (14.3%)
	$1,500 - $3,000	6 (1.5%)
	> $3,000	3 (0.8%)
Food security level	High food security	231 (57.8%)
	Low food security	105 (26.3%)
	Very low food security	64 (16%)
PHQᵇ score	Non-depressed	170 (42.5%)
	Depressed	230 (57.5%)
GAD^c^ score	Normal	172 (43%)
	Anxiety	228 (57%)
PCL^d^ score	Mean ± SD	40.53 ± 20.75

^a^SD, standard deviation; ^b^PHQ score, Patient Health Questionnaire-2; ^c^GAD score, Generalized Anxiety Disorder Scale-2; ^d^PCL score, PTSD checklist for DSM-5.

The mean weight and height were 71.3 ± 15.9 kg and 164.2 ± 8.2 cm, respectively, yielding a mean BMI of 26.4 kg/m^2^; 21% of participants were classified as obese. Mean MUAC was 30.2 cm, with 7.2% of participants identified as malnourished. Finger-prick glucose testing, conducted as an exploratory measure, showed that 91.3% of participants had normal glucose levels, while 8.8% had elevated levels. Only 12.3% of participants were fasting at the time of testing (Table 1).

Mean PHQ and GAD scores were 3.2 ± 1.8 and 3.9 ± 1.8, respectively, with 57.5 and 57% meeting criteria for major depressive disorder and generalized anxiety disorder. The mean PCL score was 40.5 ± 20.8 (Table 1).

### Food security

**Figure 1:**
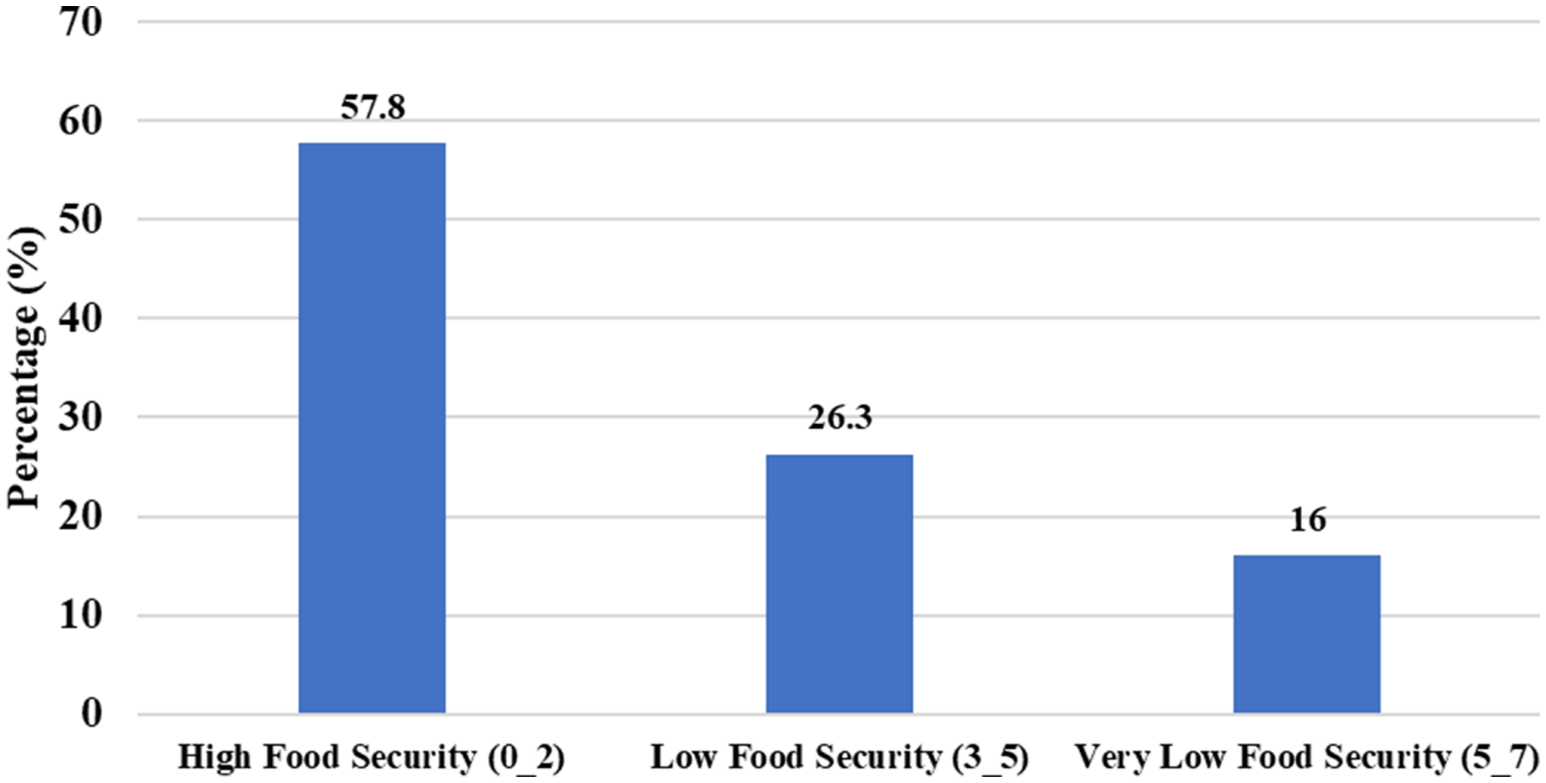
Food security level distribution.

Food security was assessed using the Arab Family Food Security Scale (AFFSS), with scores ranging from 0 to 7 (mean 2.41 ± 2.29). Overall, 28% of households were food secure, 26.3% had low food security, and 16% had very low food security (Figure 1). Common coping strategies included reducing portion sizes (35.5%), skipping meals (32.3%), and experiencing at least one day without food (20.8%). About 35.3% of participants reported that food expenditure exceeded their financial means, and 19.0% reported that household members did not eat enough due to unavailability of food (Table 2).

**Table 2:** Household food security assessment using the AFFSS (*N* = 400).

Question	Response	%
Which sentence best describes food availability?	Had enough of the kinds of food we wanted	40.3
	Had enough, but not always the kinds we wanted	41.3
	Sometimes, I did not have enough to eat	13.3
	Often did not have enough to eat	5.3
Worried about running out of food?	No	61.3
	Yes	38.8
Food bought was not enough & lacked the money to buy more?	No	64.8
	Yes	35.3
Did any family member eat less due to insufficient food?	No	81.0
	Yes	19.0
Did an adult reduce portion size due to food shortage?	No	64.5
	Yes	35.5
Did any household member skip a meal due to food shortage?	No	67.8
	Yes	32.3
Did any household member go a whole day without eating?	No	79.3
	Yes	20.8

Participants’ characteristics across the two food security categories were compared in Table 3 below. In bivariate analyses, low food security was significantly associated with female gender (*p* = 0.028), larger family size (5.46 ± 3.12 vs. 4.69 ± 2.05; *p* = 0.003), lower household income (*p* < 0.001), and higher PHQ, GAD, and PCL scores (all *p* < 0.000). The mean BRS score was 18.68 ± 2.71 for the food-insecure group and 18.18 ± 2.15 for the food-secure group (*p* = 0.045). When categorized, no statistically significant association was observed between resilience category and food security status (*p* = 0.106). Receiving psychological counseling was more prevalent among low food security participants (51.3% vs. 38.7%, *p* = 0.021). No associations were found between food security and BMI, MUAC, malnutrition, and blood glucose levels.

**Table 3:** Participants’ characteristics across different food security categories.

Variable	Category	High food security^h^ (% or Mean±SD^a^)	Low food security^h^ (% or Mean±SD^a^)	*P*-valueᶨ
Gender	Male	67.0	33.0	0.028*
	Female	54.5	45.5	
Age, years		41.91 ± 15.63	43.66 ± 15.05	0.262
Family size		4.69 ± 2.05	5.46 ± 3.12	0.003**
Number of children under 18		0.87 ± 1.68	1.15 ± 1.48	0.082
Length of displacement, days		56.90 ± 27.58	54.07 ± 40.87	0.409
Education level	No formal education	50.5	49.5	0.059
	Primary education	55.9	44.1	
	Secondary education	58.6	41.4	
	Tertiary education	69.3	30.7	
Monthly household income level	<700$	51.4	48.6	<0.000***
	700–1,500$	87.7	12.3	
	1,500–3,000$	100.0	0.0	
	≥3,000$	100.0	0.0	
BMIᵇ		26.27 ± 5.29	26.61 ± 5.64	0.536
Obesity	No	56.4	43.6	0.086
	Yes	71.4	28.6	
Blood glucose value^c^		111.78 ± 37.91	107.78 ± 27.41	0.244
	Normal (<)	57.3	42.7	0.688
	High (≥5.6 mmol/L)	60.0	40.0	
MUAC^d^		30.16 ± 5.57	30.22 ± 5.29	0.919
Malnutrition	Normal	58.5	41.5	0.284
	Malnourished	48.3	51.7	
PHQ^e^ score (Mean ± SDᵃ)		2.56 ± 1.73	3.91 ± 1.63	<0.000***
	Non-depressed	77.6	22.4	<0.000***
	Depressed	43.0	57.0	<0.000***
GAD^f^ score		2.51 ± 1.75	4.03 ± 1.77	
	No anxiety	76.7	23.3	<0.000***
	Anxiety	49.0	51.0	
PCL^g^ score		34.57 ± 19.81	48.67 ± 19.23	<0.000***
BRS^h^ score		18.68 ± 2.71	18.18 ± 2.15	0.045*
Resilience	Low	56.5	43.5	0.106
	Normal	57.4	42.6	
	High	100.0	0.0	
Received psychological counseling	No	61.3	38.7	0.021*
	Yes	48.7	51.3	
Received assistance for food	No	67.7	32.3	<0.000***
	Yes	44.0	56.0	

^a^SD, standard deviation; ^b^BMI, body mass index, classified per IOM guidelines; ^c^Blood glucose categorized using ADA (19) criteria; ^d^MUAC, mid-upper arm circumference; <23 cm indicates under-nutrition (18); ^e^PHQ score, Patient Health Questionnaire-2; ^f^GAD score, Generalized Anxiety Disorder Scale-2; ^g^PCL score, PTSD checklist for DSM-5; ^h^BRS, Brief Resilience Scale; ^i^Food security score based on AFFSS: 0–2 = high food security; 3–7 = low; *p*-values calculated using independent samples *t*-test or chi-square test as appropriate. Statistical significance set at *p* < 0.05 (*p* < 0.05 → *; *p* < 0.01 → **; *p* < 0.001 → ***).

### Prevalence of malnutrition across demographic and socioeconomic variables

Malnutrition rates were compared across population characteristics in Table 4. Malnutrition was associated with educational level (*p* = 0.048) and household income (*p* = 0.002). The highest rates were among tertiary-educated participants (11.4%) and those earning $700–1,500/month (19.3%). Food assistance reduced malnutrition prevalence (3.6% vs. 9.9%, *p* = 0.016). Moreover, high resilience scores were associated with higher malnutrition (16.7%, *p* = 0.023), but no association was found with obesity, blood glucose, or psychological counseling.

**Table 4:** Comparison of population characteristics among normal versus malnourished individuals.

Variable	Category	Normal (%)	Malnourished (%)	*P*-value
Gender	Male	96.1	3.9	0.126
	Female	91.6	8.4	
Education level	No formal education	89.9	10.1	0.048*
	Primary education	97.2	2.8	
	Secondary education	93.1	6.9	
	Tertiary education	88.6	11.4	
Monthly household income level	< 700$	94.9	5.1	0.002**
	700–1,500$	80.7	19.3	
	1,500–3,000$	85.7	14.3	
	≥ 3,000$	100.0	0.0	
Obesity	No	93.4	6.6	0.366
	Yes	90.5	9.5	
Blood glucose	Normal(<5.6 mmol/L)	92.3	7.7	0.495
	High (≥5.6 mmol/L)	97.1	2.9	
Food security score	High food security (0–2)	93.9	6.1	0.332
	Low food security (3–5)	89.4	10.6	
	Very low food security (5–7)	93.8	6.2	
PHQᵃ Score	Normal	92.4	7.6	0.792
	Major depressive disorder	93.0	7.0	
GADᵇ score	Normal	90.7	9.3	0.169
	Generalized Anxiety Disorder	94.3	5.7	
BRS^c^ Score	Low	87.9	12.1	0.023*
	Normal	95.2	4.8	
	High	83.3	16.7	
Psychological counseling during displacement	No	91.6	8.4	0.203
	Yes	95.6	4.4	
Assistance or support regarding food	No	90.1	9.9	0.016*
	Yes	96.4	3.6	

^a^PHQ score, Patient Health Questionnaire-2; ^b^GAD score, Generalized Anxiety Disorder Scale-2; ^c^BRS, Brief Resilience Scale.

*p*-values calculated using independent samples *t*-test or chi-square test as appropriate. Statistical significance set at *p* < 0.05 (*p* < 0.05 → *; *p* < 0.01 → **; *p* < 0.001 → ***).

### Logistic regression analysis of predictors for FI

Stepwise logistic regression identified several significant predictors of FI. Variables with *p* < 0.2 in bivariate analysis were included in the model. Table 5 shows the initial logistic regression model including all significant predictors. Lower monthly household income was strongly associated with higher odds of FI (OR = 0.208, 95% CI: 0.087–0.493, *p* < 0.001). Higher depression scores (PHQ) were also associated with increased odds of FI (OR = 2.124, 95% CI: 1.215–3.715, *p* = 0.008), as were higher anxiety scores (GAD) (OR = 1.921, 95% CI: 1.082–3.387, *p* = 0.025), and higher PTSD scores (PCL) (OR = 1.024, 95% CI: 1.009–1.038, *p* = 0.001). Additionally, receiving assistance or support related to food was significantly associated with FI (OR = 1.672, 95% CI: 1.013–2.759, *p* = 0.044), and larger family size showed a modest association (OR = 1.107, 95% CI: 1.001–1.223, *p* = 0.047).

**Table 5:** Stepwise logistic regression analysis - variables in the equation.

Step	Variable	OR	95% C.I. for Exp(B) (Lower - Upper)	*p*-value
Step 1a	Gender	1.355	0.784–2.342	0.276
	Family size	1.107	1.001–1.223	0.047*
	Number of children under 18	1.041	0.908–1.196	0.557
	Education level	1.091	0.869–1.369	0.453
	Monthly household income level	0.208	0.087–0.493	0.000***
	PHQᵃ	2.124	1.215–3.715	0.008***
	GAD^c^	1.921	1.082–3.387	0.025*
	PCLᵇ score	1.024	1.009–1.038	0.001***
	BRS^a^ score (1)	1.278	0.687–2.377	0.438
	Psychological counselling during displacement	0.987	0.587–1.661	0.961
	Assistance or support regarding food	1.672	1.013–2.759	0.044*
	Constant	0.018	-	0.006***

^a^ePHQ score, Patient Health Questionnaire-2 (depression); ^b^PCL score, PTSD checklist for DSM-5; ^c^GAD score, Generalized Anxiety Disorder Scale-2; ^d^BRS, Brief Resilience Scale.

Step 1, depression; Step 2, monthly household income level; Step 3 = PTSD (PCL) score; Step 4, anxiety; Step 5, assistance or support regarding food.

*Significance levels: *p < 0.05, **p < 0.01; ***p < 0.001.

Table 6 presents the stepwise logistic regression model. In step 1, depression (PHQ score) emerged as a notable predictor (OR = 4.596, *p* < 0.001). Step 2 incorporated monthly household income, which remained significantly associated with FI (OR = 0.159, *p* < 0.001), while depression maintained its significance (OR = 4.190, *p* < 0.001). In step 3, PTSD (PCL score) was added and significantly associated with FI (OR = 1.025, *p* < 0.001), with income (OR = 0.185, *p* < 0.001) and depression (OR = 3.160, *p* < 0.001) remaining significant. Step 4 included anxiety (GAD score), which showed a significant association (OR = 1.893, *p* = 0.021), while income (OR = 0.181, *p* < 0.001), depression (OR = 2.443, *p* = 0.001), and PTSD (OR = 1.021, *p* = 0.002) remained significant. In step 5, receiving food assistance was introduced and associated with higher odds of FI (OR = 1.732, *p* = 0.026), with income (OR = 0.224, *p* < 0.001), depression (OR = 2.099, *p* = 0.008), anxiety (OR = 1.864, *p* = 0.026), and PTSD (OR = 1.023, *p* = 0.001) maintaining significance. Overall, lower household income, higher depression, anxiety, PTSD scores, and receipt of food assistance were independently associated with increased odds of food insecurity.

**Table 6:** Logistic regression analysis of FI.

Step	Variable	OR	95% C.I. for Exp(B) (Lower - Upper)	*p*-value
Step 1a	PHQᵃ	4.596	2.945–7.175	<0.001***
Step 2b	Monthly household income level	0.159	0.070–0.358	<0.001***
	PHQᵃ	4.190	2.644–6.639	<0.001***
Step 3c	Monthly household income level	0.185	0.082–0.418	<0.001***
	PHQᵃ	3.160	1.949–5.122	<0.001***
	PCLᵇ score	1.025	1.012–1.037	<0.001***
Step 4d	Monthly household income level	0.181	0.079–0.414	<0.001***
	PHQᵃ	2.443	1.439–4.150	0.001**
	GAD	1.893	1.099–3.260	0.021*
	PCLᵇ score	1.021	1.008–1.034	0.002**
Step 5e	Monthly household income level	0.224	0.097–0.513	<0.001***
	PHQᵃ	2.099	1.214–3.629	0.008**
	GAD^c^	1.864	1.079–3.220	0.026*
	PCLᵇ score	1.023	1.010–1.037	0.001**
	Assistance or support regarding food	1.732	1.067–2.811	0.026*

^a^PHQ score, Patient Health Questionnaire-2 (depression); ^b^PCL score, PTSD checklist for DSM-5; ^c^GAD score, Generalized Anxiety Disorder Scale-2 (anxiety).

Step 1, depression; Step 2, monthly household income level; Step 3, PTSD (PCL) score; Step 4, Anxiety; Step 5, assistance or support regarding food.

*Significance levels: *p < 0.05, **p < 0.01, ***p < 0.001.

## Discussion

This cross-sectional study was conducted among displaced families during Lebanon’s September–December 2024 war. During this war, the number of displaced persons was more than 1.5 million, with 900,000 internally displaced, and around 640,000 moving to Syria (29). The novel findings from this study were that household income, GAD, major depressive disorders, and PTSD were associated with FI. Moreover, % of FI was 42.4%, with 16.1% being very low FI, while only 28% were food secure. It is noteworthy to mention that the % of mental health problems, such as GAD and major depressive disorders, was high at 57.5 and 57%, respectively. Moreover, the mean PCL score was 40.53, indicating high PTSD levels. The data also highlights the prevalence of obesity (21%) in conjunction with under-nutrition (7.2%), suggesting a dual burden of malnutrition.

The high prevalence of FI is comparable to that published by the IPC right after the war and with the food insecurity prevalence reported before the war (30, 31). However, an apparent deterioration of the situation and higher FI prevalence can be seen when compared to the pre-economic crisis data. Moreover, our data shows less severe rates than similar conflicts in Syria and Yemen (32).

Gender appears as an essential determinant of FI (*p* = 0.028), and women were more affected than men, reflecting a context of economic marginalization, limited access to employment, and decreased capital compared to men (33). Moreover, in displaced families, women are reduced to caregiving roles, where they prioritize their children’s nutrition (34).

A bigger family size was associated with higher FI (*p* = 0.003), which aligns with previous studies (35, 36). The increase in household numbers, primarily dependent members such as children or the older adults, can increase demand for food, adding more economic burden on the already limited resources in displacement settings (9). Moreover, 83.3% of households have a monthly income of less than $700$, further exacerbating this issue among big households.

While educational level has been shown to be associated with FI (37), our study did not prove this association (*p* = 0.059). Indeed, better education is associated with greater employment opportunities and better health literacy, which could represent a protective factor against FI (37). The non-significance in this study could indicate that this relationship could be undermined in a conflict context, and even highly educated people could face systemic and conflict-related barriers.

Unsurprisingly, it was found in this study that household income was a significant predictor of FI (*p* < 0.001). Displacement can disrupt income-generating activities and lead to asset depletion, leaving families highly dependent on humanitarian aid, which is often insufficient or inconsistent (38, 39).

Interestingly, the relationships identified in this analysis—income, household size, and gender as determinants of food insecurity—must be interpreted within the broader conflict and displacement context. Displacement disrupts income flows, depletes assets, and reshapes household composition, thereby exacerbating vulnerability. For example, women’s increased susceptibility to food insecurity is intensified by exclusion from formal labor markets and concentration in unpaid caregiving roles, particularly in displacement situations with scarce opportunities (39). Larger households, already burdened with higher consumption requirements, face more severe constraints when housing is precarious and incomes are unstable or absent. The loss of livelihoods, dependency on often irregular humanitarian aid, and structural exclusion from employment and social protection systems further amplify the economic exclusion of displaced families (40). During Lebanon’s 2024 crisis, these vulnerabilities were worsened by the ongoing financial collapse and hyperinflation (41), rendering displaced families particularly disadvantaged compared to non-displaced populations. Therefore, displacement should not be regarded merely as background context but as a structural determinant that intensifies conventional socio-demographic drivers of food insecurity.

As for mental health variables, our study identified a strong association between FI and poor conditions of mental health, in particular depression, anxiety, and PTSD. Food insecurity victims were reporting distress with a prevalence of 57 and 51% for major depressive disorder and major anxiety disorder, respectively, which was associated with FI (*p* < 0.001), showing the psychosocial dimension of FI. The logistic regression analysis integrating the mental health variables identified depression, anxiety, and PTSD as significant predictors for FI with ORs equal to 2.1, 1.86, and 1.02, respectively. Numerous studies have supported this relationship (41–45). For instance, in two different studies by Garg et al. (46) and Hernandez et al. (47), maternal depression seems to predict household food insecurity (OR = 1.5 and 2.03, respectively). Similarly, Whittle et al. (48) showed this association with anxiety and PTSD. Previous reports showed that mental health distress could lead to reduced productivity and significant impairment in different cognitive domains (49). In addition, such a mental condition could constitute a cognitive load and impair decision-making capabilities, reducing searching and benefiting from aid systems (18). It is noteworthy to consider that this link is more likely bidirectional; however, in a cross-sectional design, the time dimension is nonexistent, which limits the ability to determine whether the identified factors preceded the occurrence of FI.

On the other hand, BRS was not associated with FI. This result contradicted the studies by Smith et al. (50) and d’Errico et al. (51), where higher resilience predicts more food security. However, BRS could be limited in a conflict context and may not capture context-specific resilience. Additionally, the Brief Resilience Scale may not fully capture context-specific coping in displacement settings, limiting its association with FI (52).

The data also suggests that assistance in food was not successful in reducing FI, as 56% of aid recipients remained food insecure. Households receiving aid had 1.7 times higher odds of food insecurity (OR = 1.732, *p* = 0.026), suggesting inconsistency and insufficiency in aid amounts. However, food assistance reduced malnutrition prevalence by 6.3 percentage points (9.9% → 3.6%, *p* = 0.016). A previous study, in a more complex context in Mali among internally displaced persons, showed that food assistance had a protective effect on food expenditure, consumption, and micronutrient availability (53). Out of conflict context, studies also emphasized the efficient role of food aid in reducing food insecurity and escaping hunger (54, 55).

No significant association was found between food insecurity (FI) and malnutrition, BMI or MUAC. This may reflect the low prevalence of undernutrition in the study population, the short average displacement duration (56 days), or the slow response of adult anthropometry to short-term FI. These null findings align with the heterogeneous literature on the “food insecurity–obesity paradox” (56–59), where the relationship between FI and nutritional outcomes remains inconsistent. Methodological limitations such as measurement error and reporting bias may also contribute to these results.

Additionally, the coexistence of malnutrition (7.2%) and obesity (21%) among displaced families was observed, though no statistically significant association between malnutrition and FI was detected (*p* = 0.284). This dual burden may reflect the ongoing economic crisis in the country since 2019, during which many families have increasingly relied on cheap, calorie-dense, and nutrient-poor foods due to hyperinflation and dependence on food assistance baskets (60). Furthermore, mental health distress may have influenced dietary behaviors, such as appetite loss or stress-induced eating, as reported in other contexts (61).

Taken together, these findings highlight the significant change that might occur in post-conflict Lebanon. The interplay between food insecurity and mental health emphasizes the need for the employment of new intervention strategies that provide nutritional assistance with accessible psychological services, particularly for larger households, female-headed families, and those experiencing high levels of distress. Moreover, our findings show an urgent re-evaluation of the quality of food aid in terms of consistency and nutritional sufficiency. The alignment of mental health and food security interventions could present a more sustainable approach to breaking the cycle of economic hardship, stress, and inadequate diet in displacement settings. Future research could help clarify whether poor mental health precipitates food insecurity, or vice versa, and pinpoint the most effective forms of support in this context.

### Strengths and limitations

This study provides important insights into the FI faced by displaced families during Lebanon’s 2024 war, highlighting several socio-economic and psychological factors that contribute to the problem. One key strength of the study is its real-world context, offering up-to-date data from a region experiencing severe conflict. It also covers various determinants, including socio-demographics, mental health, and food insecurity status, giving a holistic view. However, the study’s cross-sectional design limits the ability to draw causal conclusions about the relationship between these factors. Additionally, the narrow sampling frame is a limitation. Indeed, among the 1.5 million displaced persons, only those residing in displacement centers were included in the study, excluding individuals who are renting houses and those who left the country. This limits the generalizability of the findings. Another limitation is the potential bias in self-reported data, especially concerning mental health and FI, which could lead to underreporting or social desirability biases. The study also identifies the inefficacy of food assistance in reducing FI, suggesting issues with the consistency and sufficiency of aid. It should also be noted that glucose testing was exploratory in nature and yielded no significant findings. As such, it was not integrated into the primary study aims or subjected to detailed analysis.

## Conclusion

In conclusion, while the study offers valuable insights into the complexities of FI in displacement contexts, further research is needed to explore causal relationships and assess the effectiveness of interventions. Policymakers should consider integrating mental health support alongside food aid to address the multifaceted nature of food insecurity in conflict settings.

## Data Availability

The raw data supporting the conclusions of this article could be made available by the authors upon reasonable request. The full database cannot be shared openly for confidentiality and anonymity reasons. Requests of data sharing can be addressed to the first or corresponding authors.
